# A Meta-Analysis Methodology in Stan to Estimate Population Pharmacokinetic Parameters from Multiple Aggregate Concentration–Time Datasets: Application to Gevokizumab mPBPK Model

**DOI:** 10.3390/pharmaceutics16091129

**Published:** 2024-08-27

**Authors:** Evangelos Karakitsios, Aristides Dokoumetzidis

**Affiliations:** Department of Pharmacy, National and Kapodistrian University of Athens, Panepistimiopolis Zografou, 15784 Athens, Greece

**Keywords:** aggregate concentration-time data, population pharmacokinetic parameters, gevokizumab, RStan, meta-analysis

## Abstract

The aim of the present study was to develop and evaluate the performance of a methodology to estimate the population pharmacokinetic (PK) parameters along with the inter-individual variabilities (IIVs) from patients’ reported aggregate concentration–time data, in particular, mean plasma concentrations and their standard deviations (SDs) versus time, such as those often found in published graphs. This method was applied to the published data of gevokizumab, a novel monoclonal anti-interleukin-1β antibody, in order to estimate the drug’s population pharmacokinetic (PopPK) parameters of a second-generation minimal physiologically based pharmacokinetic (mPBPK) model. Assuming this mPBPK model, a mixed effects approach was utilized to allow accounting for the random inter-group variability (IGV) that was assumed among different dosage groups. The entire analysis was performed using R software (Rstudio) and the Bayesian software tool RStan was used for the application of Bayesian priors on the parameters. Conclusively, the proposed method could be applied to monoclonal antibodies for which the second-generation mPBPK model has been proposed as well as to other drugs with different PK models when only a published graph with aggregate concentration–time data is available. In addition, the method could be used when multiple aggregate datasets from different sources need to be combined in a meta-analysis approach in order to estimate the PopPK parameters of a drug.

## 1. Introduction

Pharmacokinetic (PK) profiles depicting drug plasma concentrations versus time are often available in the literature in aggregate form, with average concentrations and respective standard deviations from several subjects. These often appear in graphs showing average profiles with error bars. Given that this aggregation of data encloses valuable information to the medicinal community, it would be desirable to develop a methodology, in the context of population pharmacokinetics, to estimate population pharmacokinetic (PopPK) parameters along with the inter-individual variabilities (IIVs) from these published data in order to possibly recover the loss of information from averaging.

Cavelti-Weder et al. have published their work concerning gevokizumab’s PK profile in type 2 diabetes patients in the form of such a graph [1]. Gevokizumab is an investigational drug, a unique inhibitor of interleukin-1b (IL-1b) signaling that may offer an alternative to current therapies for IL-1b–associated autoinflammatory diseases [2]. It has been proposed that by decreasing the potency of IL-1β, while allowing binding to its clearance and inhibitory receptors, gevokizumab treatment will attenuate IL-1β activity in concert with endogenous regulatory mechanisms [3]. Currently, a phase Ib study of gevokizumab is carried out. This study will determine the pharmacodynamically active dose of gevokizumab and its tolerable dose as well as the preliminary efficacy of the drug in combination with its standard of care anti-cancer therapy in patients with metastatic colorectal cancer, metastatic gastroesophageal cancer, and metastatic renal cell carcinoma [4]. It is noted that all the previous diseases are directly associated with IL-1β [5,6,7]. Furthermore, gevokizumab has shown promising results in early clinical trials for inflammation-mediated diseases such as uveitis due to Behçet disease and autoimmune non-necrotizing anterior scleritis [8]. Also, no serious adverse events have been reported in relation to gevokizumab use. Of note, no serious infections have been reported. The most common reported side effects include upper respiratory tract infection, headache, and dizziness [9].

In addition, for gevokizumab, a second-generation minimal physiologically based pharmacokinetic (mPBPK) model has been proposed [10]. mPBPK models offer a simple and sensible modeling approach to incorporate physiological elements into PK analysis when only plasma data are available [11]. The second-generation mPBPK model was developed specifically for linear monoclonal antibody PK analysis. Two groups of tissues (tight and leaky) are defined in the model and only three parameters need to be estimated for gevokizumab: its vascular reflection coefficients (rc_1_ and rc_2_) and its clearance applied to plasma. Cao et al. estimated these three parameters for gevokizumab based on the drug’s mean plasma concentrations versus time, considering that these means represent a “mean” patient [10]. This approach, however, does not consider each patient’s variability.

Furthermore, the drug-related parameters of a PBPK model can be estimated with various methods based on the clinical data [12]. One such method is Bayesian inference, where our prior knowledge is updated with the clinical data, resulting in new estimated parameters in the form of posterior distributions. One main advantage of the Bayesian approach is that if the Bayesian framework is combined with hierarchical modeling, then the model can describe both the individual and population levels [13,14]. Concerning the methodology we wish to develop, in the case that a range of doses is available, the individual levels refer to each different dosage group, while the population levels refer to all dosage groups together. In that way, the inter-group variabilities (IGVs) are implied.

Moreover, Bayesian inference is usually conducted through Markov Chain Monte Carlo (MCMC) [15]. Hamiltonian Monte Carlo (HMC) is the most known non-random walk MCMC—a main group of MCMC algorithms. Stan is a newer free software platform for performing Bayesian inference that was released in 2012 and the default sampler in Stan is the No-U-Turn Sampler (NUTS), which is an adaptive variant of HMC [16,17,18]. Stan has been applied in various areas, including social sciences, finance, pharmacometrics, and Pharmacokinetic–Pharmacodynamic (PK–PD) modeling [19]. Also, Stan has recently been used to estimate the drug-related parameters of a whole-body PBPK model with a hierarchical Bayesian framework [20]. Finally, to our knowledge, a Bayesian aggregation of average data has been made using Stan to model jointly the individual data of the patients and external aggregate data in a meta-analysis approach [19]. In this approach, however, the average data that are utilized are only in the form of means and do not include standard deviations (SDs).

## 2. Materials and Methods

Initially, only aggregate datasets in the form of mean plasma concentrations and their corresponding SDs versus time were assumed to be available for the kinetics of a drug, probably taken from the literature. Several such datasets referring to different dosage-groups may be available. The pursuit of the present work is to develop a methodology to estimate the PopPK parameters of gevokizumab after single intravenous (IV) doses of the drug from the patients’ reported aggregate datasets [1]. Our work concerning gevokizumab, whose PK is assumed to follow a second generation mPBPK model, is separated into two parts. (i) A simulation study was performed to evaluate the performance of the methodology applied to the aggregate simulated data of only one dosage group. In this study, various modelling choices were tested to assess the overall limitations of the methodology. (ii) Application to the real gevokizumab datasets was carried out. In the second part, the results of the previous simulation study were applied to the real gevokizumab datasets to estimate the drug’s PopPK parameters. Initially, the PopPK parameters of gevokizumab were estimated for one single dosage-group and then a mixed effects approach was used to utilize all available dosage group’s data. All simulations were performed with R version 3.5.1 (Rstudio version 1.2.5001) and the PopPK parameters were estimated in RStan version 2.19.2.

### 2.1. Clinical Data of Gevokizumab

The available aggregate data that were used for the latter application to the real gevokizumab datasets refer to 6 different groups of patients with type 2 diabetes, which have been described in a previous study [1]. Each group consisted of 10 patients, apart from one group that consisted of 5 patients. The patients of all the groups received a single IV dose of gevokizumab. The doses for the first 5 groups were 0.01, 0.03, 0.1, 0.3, and 1.0 mg/kg, while the dose for the 6th group was 3.0 mg/kg. Following the administration of the drug, plasma samples were collected at 4, 8, 24, 48, 72, 96, 168, 216, 264, 336, 504, 672, 1008, and 1344 h. Mean plasma concentrations as well as their SDs versus time for gevokizumab were captured using Digitizer software.

### 2.2. Simulation Study

Initially, mean plasma concentrations and SDs of gevokizumab were simulated with the R package deSolve based on the above differential equations of the mPBPK model. deSolve is a solver for initial value problems of differential equations [21]. Concentrations from a Monte Carlo (MC) simulation, for a number of patients, were generated with this package at the identical time points to those of the sampling scheme in the study of Cavelti-Weder et al., including the time point 0 [1]. For the simulation, the following steps were followed. The PK parameters of plasma clearance and volume of human body of these patients were considered to be independently, lognormally distributed. Additionally, IIV was not applied to the two reflection coefficients since the obtained SDs with this application in our simulation study deviated a lot from the SDs of the real clinical data of gevokizumab. It is noted that the same IIV term for volume, namely, the SD of the lognormal distribution of volume of human body, is applied to all 4 volumes of the mPBPK model in our analysis. Our concept is that a bigger patient will obviously have more plasma and tight and leaky tissues as well as larger lymph volumes than a smaller one. Afterwards, the concentrations at each time point were calculated for all patients with the package deSolve and an exponential residual variability term was assumed for every one of these concentrations. Finally, the mean plasma concentrations and their SDs versus time were obtained.

Next, the plasma concentrations versus time of a large number of virtual patients were generated in Stan from a MC simulation from the distributions of the mPBPK parameters. These patients essentially represent one dosage group’s virtual population. The PK parameters of plasma clearance and volume of human body of these individuals were also considered to be independently, lognormally distributed, in accordance with the respective parameters used in the simulated data. Conclusively, our model was parameterized in terms of the vascular reflection coefficients rc_1_ and rc_2_ for tight and leaky tissues, respectively, the mean drug plasma clearance CL_p_, and the IIV terms ω_CLp_ and ω_V_ for the SD of the lognormal distributions of plasma clearance and volume of human body, respectively. Also, two separate exponential residual error terms were assumed, one for the means (sigma_1) and one for the SDs (sigma_2).

Since our system of differential equations is linear, the matrix exponential method was used to solve it in Stan [22]. This method uses a Pade approximation coupled with scaling and squaring [23]. Additionally, the priors placed on the parameters of our model were as non-informative as possible. In particular, the weakly informative prior of half-normal (0,1) was placed on the two exponential residual error terms (sigma_1 and sigma_2) as well as on the two exponential IIV terms (ω_CLp_ and ω_V_). It is noted that, regarding all the exponential terms, all the values sampled from the half-normal (0,1) prior distribution are positive and decrease slowly from 0, allowing large values being sampled with much lower probability. Also, the weakly informative prior of half-normal (0,1) was placed on the parameter of mean plasma clearance, considering that the estimation of gevokizumab’s plasma clearance by Cao et al. was 0.00668 L/h [10]. Moreover, a prior of beta (1,1) was placed on the two vascular reflection coefficients to ensure both their lower bound of 0 as well as their upper bound of 1.

As far as the number of virtual patients used in Stan is concerned, our goal was to use 1000 for the Monte Carlo step so that the means and SDs obtained each time are independent of the patients. However, it was found that solving the differential equations for 1000 patients was very computationally expensive. To overcome this, Latin Hypercube Sampling (LHS) was used for the MC step to improve speed. LHS is a statistical method for generating a near-random sample of parameter values from a multidimensional distribution. The sampling method is often used for Monte Carlo integration and was described by Michael McKay in 1979 [24].

Moreover, the random values of the MC simulation generated in Stan were chosen to be the same ones at each iteration of the algorithm. As a result, the values of plasma clearance ($\xi_{{CL}_{p}}$) and human volume ($\xi_{V},$ where V is either $V_{plasma}$, $V_{lymph}$, $V_{tight}$ or $V_{leaky}$) of each virtual i-patient were as described in the following Equations (1) and (2):

(1) $$\xi_{{CL}_{p}\;(i)}={CL}_{p}*exp\left({\eta^{\prime }}_{{CL}_{p}}*\omega_{CLp}\right)\;\mathrm{where}\;{\eta^{\prime }}_{{CL}_{p}}\sim Ν\left(0,1\right)$$

(2) $${\;\xi }_{V\;(i)}=V*exp\left({\eta^{\prime }}_{V}*\omega_{V}\right)\;\mathrm{where}\;{\eta^{\prime }}_{V}\sim Ν(0,1)$$

Instead of producing 1000 random values for ${\eta^{\prime }}_{{CL}_{p}}$ that follow a normal distribution with mean 0 and variance 1 as well as 1000 respective random values for ${\eta^{\prime }}_{V}$, according to the Equations (1) and (2), a smaller number of these was chosen to be produced with LHS. To define this specific number, the sampling distributions of the means as well as of the SDs were generated, by repeated sampling (100,000 samples), in two cases: the case of 1000 random values being sampled with normal sampling and the case of an increasing number of random values being sampled with LHS. Eventually, it was found that 60 random values with LHS are required so that the standard error (SE) of the means as well as the SE of the SDs are lower than the respective SEs with normal sampling. Also, the package “lhs” in R was used for this LHS step [25].

Furthermore, for each time point, the mean plasma concentration and its corresponding SD were obtained. These aggregate data were finally fitted in Stan to the respective simulated dataset. The model on the platform of Stan was run with 4 chains with 1000 samples each and used 1000 warm-up iterations. It is noted that these are the default values used in Stan. In addition, the default initialization was followed. The model was run on a computer with an Intel Core i7-3770 (3.4 GHz), 12 GB RAM and windows 10 OS.

To investigate the performance of the method under different circumstances, 2 dataset scenarios were considered; namely, dataset I, with a number of individuals (N) equal to 24 and 5% residual variability (rv), from which the simulated aggregate data were derived; and dataset II, with N = 24 and rv = 10%. It is noted that the number 24 is used since it is a typical number of patients in a clinical trial, while residual variability was added onto the plasma concentrations as noise. Moreover, to evaluate the application of the proposed method to gevokizumab’s real clinical data, another dataset scenario was considered (dataset III) with N = 10 and rv = 5%. As discussed below, 10 is the number of patients in the study from which the real clinical data of gevokizumab was digitized [1]. Also, concerning the residual variability, an exponential model was used in all three cases.

Firstly, to give a better insight into our proposed methodology’s objective and to visually compare the available aggregate data with the respective predictions of our model, a plot was generated concerning dataset scenario I. The simulated aggregate data, for dataset I, were generated and used in Stan to obtain the PopPK parameters. Then, these parameters were used to simulate the plasma concentrations versus time of 1000 random individuals. For each time point, the mean plasma concentrations as well as their corresponding SDs versus time were obtained. Finally, the simulated aggregate data as well as our model’s predictions, in the form of colored areas which represent the predicted mean plasma concentrations with their SDs (upper and lower bound of the areas), were depicted in the plot.

Afterwards, a representative visual predictive check (VPC) was performed for every one of the 3 dataset scenarios. Each time, the 5th, 50th, and 95th percentiles of the simulated data (original dataset) were calculated, and the parameter estimates obtained by fitting the model to the original dataset were then used to simulate 1000 datasets of the same form as the original one. A non-parametric 90% prediction interval around the median of each simulated dataset was constructed to calculate the 5th, 50th, and 95th percentiles of the model predictions. Also, 95% confidence intervals (CI) were calculated around each of these percentiles from the 1000 replicates. Finally, to visually compare the prediction of the model to the original dataset, it was checked whether the percentiles of the original dataset fell within the 95% CI of the respective percentiles of the model predictions.

To further evaluate the performance of the method and its limitations, simulations and estimations were carried out. For each scenario, 200 simulated aggregate datasets were generated, from which the accuracy and precision for estimating the PopPK parameters were calculated. The PopPK parameters were estimated according to the process described above. The dose for this simulation study was 7 mg (one of the doses used in the study of Cavelti-Weder et al. in 2012 adapted to a 70 kg body weight person). The parameters used for the simulations are presented in Table 1. The mean population parameters of gevokizumab were identical to the ones estimated by Cao et al. in 2013, while the IIV terms were decided to be equal to 20%.

As mentioned above, the performance of the method was evaluated by calculating bias and precision. The percent mean relative bias (RBIAS), was calculated as follows in Equation (3):

(3) $$\%\;RBIAS=\frac{100}{n}*\sum_{i=1}^{n}\frac{\theta_{E_{i}-}\theta_{T_{i}}}{\theta_{T_{i}}}$$

where n is the number of datasets that were generated, $\theta_{E_{i}}$ is the estimated value of each PK parameter for the ith dataset, and $\theta_{T_{i}}$ is the value of the same parameter generated in the respective simulation of the ith dataset—not the nominal values presented in Table 1. Note that parameters θ can be either the mean PopPK parameters or the IIV terms. The precision was calculated as the percent relative root mean squared error (RMSE) and as the percent relative absolute error (RAE), respectively, as shown in Equations (4) and (5):

(4) $$\%\;RMSE=100*\sqrt{\frac{1}{n}*\;\sum_{i=1}^{n}{\left(\frac{\theta_{E_{i}-}\theta_{T_{i}}}{\theta_{T_{i}}}\right)}^{2}}$$

(5) $$\%\;RAE=\frac{100}{n}*\sum_{i=1}^{n}\frac{{|\theta }_{E_{i}-}\theta_{T_{i}}|}{\theta_{T_{i}}}$$

### 2.3. Second-Generation mPBPK Model for Gevokizumab

The model structure of the second-generation mPBPK is shown in the Supplementary Materials (Figure S1), as described by Cao et al. [10]. It is noted that two groups of tissues are defined in the model according to their vascular endothelial structure, namely, tight and leaky tissues. Tight tissues have continuous capillaries, while leaky tissues have discontinuous or fenestrated capillaries. Tight tissues include muscle, skin, adipose, and brain, and leaky tissues refer to all other tissues (liver, kidney, heart, etc.). For gevokizumab, it has been found that the model that assumes clearance from plasma describes better the PK of the drug. The differential equations for this mPBPK model are provided in the Supplementary Materials (Equations (S1)–(S4)).

### 2.4. Application to the Real Datasets of Gevokizumab

The same process followed in the previous simulation study was applied to the real gevokizumab datasets, except for the fact that the real aggregate data, which were captured using Digitizer software, were used instead of the aggregate simulated data, which were generated with the package deSolve, to estimate the drug’s PopPK parameters.

Initially, the PopPK parameters of gevokizumab were estimated for one single dosage group, in particular, for the 7 mg dosage group. The same sampling scheme that was used in the study of Cavelti-Weder et al. was also applied and mean plasma concentrations along with their SDs at these time points (clinical data) were captured using Digitizer software. The respective predictions generated in Stan were then fitted to these clinical data to estimate the PopPK parameters. It is noted that this dosage group consisted of 10 patients.

Further, since different dosage groups were available, the Bayesian framework described above was combined with hierarchical modeling and a mixed effects approach was used to utilize most available dosage group’s data. In the study of Cavelti-Weder et al. in 2012, there were 5 dosage-groups that consisted of 10 patients (0.01, 0.03, 0.1, 0.3, and 1.0 mg/kg) and 1 group that consisted of only 5 patients (3.0 mg/kg). In our study, only the 5 groups with 10 individuals were included in the analysis since the number of 5 was considered too low to obtain accurate means and SDs.

More particularly, IGV was considered for the 3 PK parameters of each group (CL_p_, σ_1_ and σ_2_), following log-Student’s t-distribution, because of the small size of the dosage-groups, estimating the respective mean group and IGV terms. The exponential model was used for the random IGVs. Also, the degrees of freedom used for the Student’s t-distribution were fixed at the fairly low value of 5. In addition, separate exponential IIV terms were assumed for each group without a distributional assumption for IGV, in a semi-hierarchical approach. Finally, two exponential residual error terms were considered, one for the means and one for the SDs.

The priors placed on the parameters of our model were as follows. A prior of half-normal (0,1) was placed on all the exponential terms, namely, the 2 exponential residual error terms (sigma_1 and sigma_2), the exponential IIV terms (ω_CLp_ and ω_V_) for each dosage-group as well as the 3 exponential IGV terms (γ_CLp_ for CL_p_, γ_rc1_ for rc_1_, and γ_rc2_ for rc_2_). A prior of half-normal (0,1) was also placed on the mean group PopPK parameter CL_p_, while a prior of beta (1,1) was placed on the two mean group vascular reflection coefficients.

The model on the platform of Stan for the single dosage group was run with 4 chains with 1000 samples each and used 1000 warm-up iterations, while the model for all dosage-groups was run with 4 chains with 2000 samples each and used 2000 warm-up iterations.

Furthermore, to emphasize the potential use of our methodology to a variety of drugs and PK models, a template is provided in the Supplementary Materials. In this template, the method was applied to simulated data of a theoretical drug that was assumed to be administered in the form of a simple IV bolus and a one-compartment open model was utilized.

## 3. Results and Discussion

### 3.1. Gevokizumab’s Simulation Study

**Figure 1:**
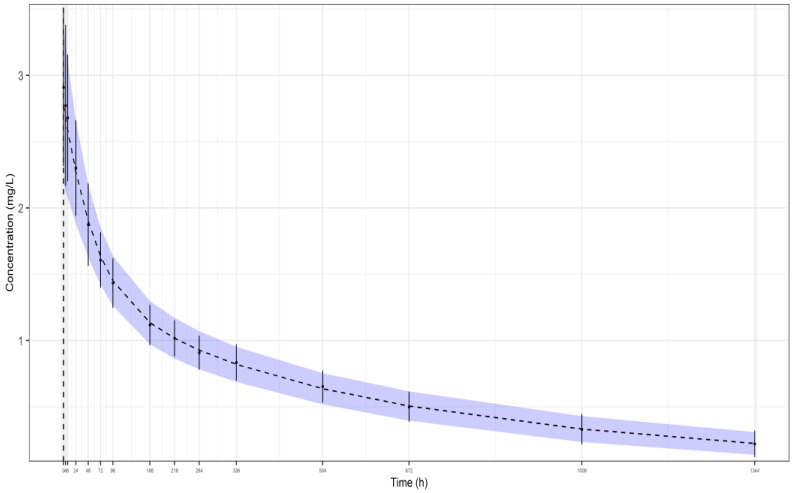
Plot of our model’s predicted means and SDs (1000 random patients) after fitting our model to the simulated aggregate data of a representative simulated study from dataset I (24 individuals, 5% residual variability). The following model parameter estimates were used: CLp = 0.00657 L/h, rc1 = 0.911, rc2 = 0.872, ωCLp = 0.221, and ωV = 0.209. Key: Dashed line: predicted means, blue band: predicted SDs, black points and error bars: observed (from simulation) means and SDs, respectively.

The plot, which was, firstly, generated to give a better insight into the aim of our proposed methodology, is presented in Figure 1. The black dashed line, shown in the plot, represents the model’s predicted mean plasma concentrations of 1000 random patients, while the upper and lower bounds of the blue area reflect the model’s predicted SDs of the plasma concentrations, in particular, the (mean + SD) and (mean − SD), respectively. Also, the black dots with the error bars represent the simulated aggregate data, namely, the means and SDs, generated with the package deSolve. As shown in the plot, the model seems to be able to predict the simulated aggregate data. The respective parameter estimates are shown in the legend of Figure 1.

**Figure 2:**
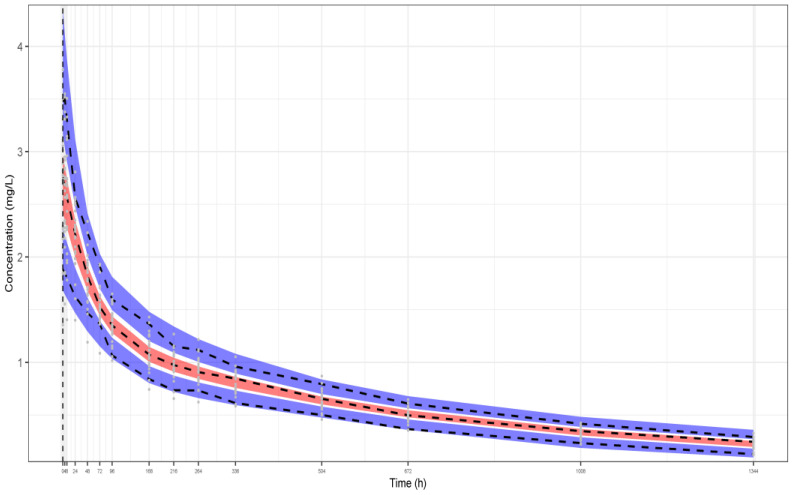
VPC of our model fitted to the aggregate data of a representative simulated study from dataset I (24 individuals, 5% residual variability). The following model parameter estimates were used: CL_p_ = 0.00651 L/h, rc_1_ = 0.929, rc_2_ = 0.830, ω_CLp_ = 0.136, and ω_V_ = 0.212. Key: 3 bands are 95% CIs of 95th, 50th and 5th percentiles of predictions, respectively from top to bottom, dashed lines are the respective percentiles of the observations (from the simulated data).

**Figure 3:**
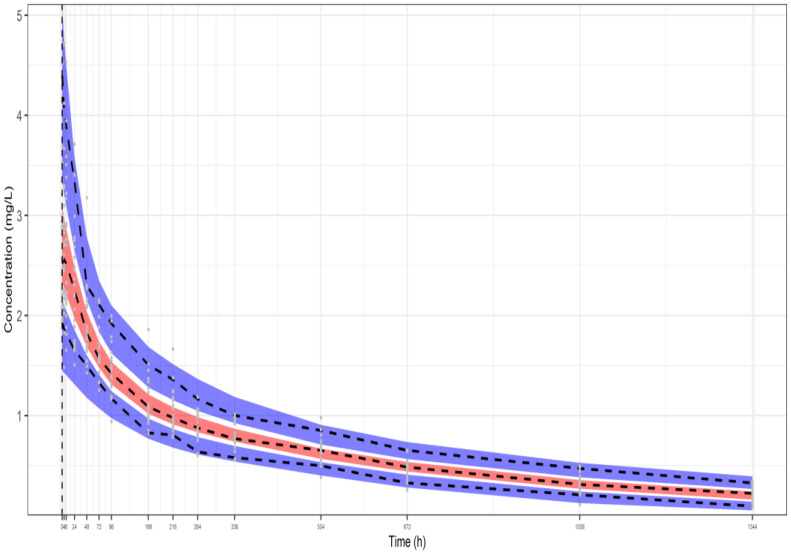
VPC of our model fitted to the aggregate data of a representative simulated study from dataset II (24 individuals, 10% residual variability). The following model parameter estimates were used: CL_p_ = 0.00673 L/h, rc_1_ = 0.928, rc_2_ = 0.861, ω_CLp_ = 0.190, and ω_V_ = 0.285. Key: 3 bands are 95% CIs of 95th, 50th and 5th percentiles of predictions, respectively from top to bottom, dashed lines are the respective percentiles of the observations (from the simulated data).

**Figure 4:**
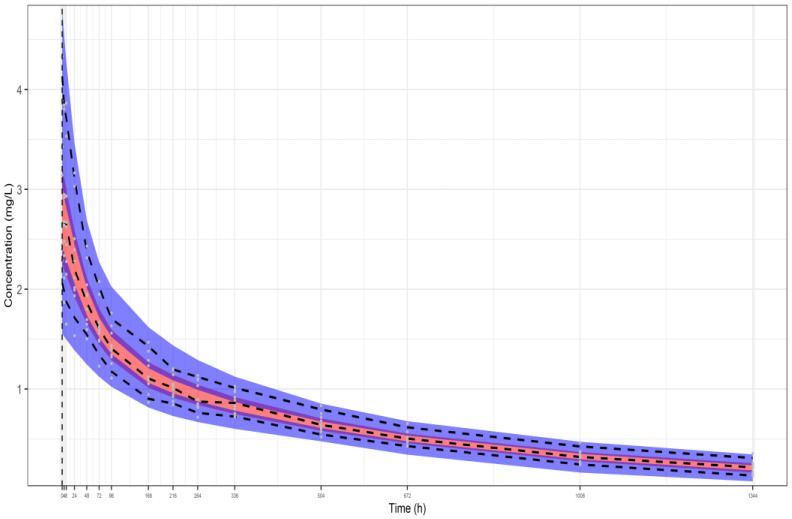
VPC of our model fitted to the aggregate data of a representative simulated study from dataset III (10 individuals, 5% residual variability). The following model parameter estimates were used: CL_p_ = 0.00660 L/h, rc_1_ = 0.939, rc_2_ = 0.852, ω_CLp_ = 0.130, and ω_V_ = 0.253. Key: 3 bands are 95% CIs of 95th, 50th and 5th percentiles of predictions, respectively from top to bottom, dashed lines are the respective percentiles of the observations (from the simulated data).

The three representative VPCs that were plotted for each dataset scenario, as described above, are presented in, Figure 2, Figure 3 and Figure 4. The black dashed lines shown in these plots represent the median, the fifth, and the 95th percentile of the plasma concentrations of the original simulated dataset, while the grey dots are all the concentrations at each time point. Also, the 90% prediction intervals (fifth, 50th, and 95th percentiles from 1000 virtual patients) together with 95% CI around each of the percentiles are shown as well. The red filled areas represent the CI of the medians, while the blue areas represent the CI of the fifth and 95th percentiles. It is noted that all the data lie within the 95% CI of the predictions. The VPCs for datasets I (Figure 2) and II (Figure 3) demonstrate that the model can predict the original simulated dataset, derived from 24 patients, although the parameters have been estimated only from the aggregation of these data, namely, the mean plasma concentrations and their respective SDs. The respective parameter estimates are shown in the legends of the three Figures. In addition, the VPC for 10 patients (dataset III—Figure 4) results to a similar plot showing good predictions, with slightly wider CIs.

As mentioned in Section 2, to further evaluate the performance of the method, simulations and estimations were carried out for each dataset scenario. Then, the bias and precision of the estimates were calculated from 200 simulated datasets, as mentioned above. All options considered in this part of the simulation study are summarized in Table 2, showing the bias (% RBIAS) as well as the precision (% RMSE and % RAE) of the estimates. It is noted that not all 200 simulated datasets were used, in each of the three scenarios, to estimate the final values of % RBIAS, % RMSE, and % RAE. For each scenario, in a few of these 200 runs, either there were divergent transitions after warmup or Sample Sizes (Tail and Bulk Effective Sample Sizes) were too low. In particular, 196, 193, and 189 simulated datasets were finally used for the first, second, and third scenario, respectively. Concerning the excluded datasets, it was found that increasing the number of samples used on the platform of Stan results in a fine convergence. Hence, our method can be applied to the vast majority of the possible datasets generated in our study with the default four chains with 1000 samples each and 1000 warm-up iterations.

Moreover, as shown in Table 2, for all three different scenarios, the estimates are highly unbiased and precise apart from the IIV term ω_V_ in dataset II (24 individuals and 10% residual variability), where the values of % RBIAS, % RMSE, and % RAE are over 20%. The results show that the method can estimate all the parameters with satisfactory bias and precision when the residual variability is at a level of 5% and the aggregate data have been obtained from either 24 or 10 individuals. On the contrary, when the residual variability increases (rv = 10%), the estimates, especially the IIV terms, become more biased as well as less precise. Also, when the residual variability is equal to 5%, the increase in the number of individuals used to obtain the aggregate data produces, generally, estimates with a lower bias and higher precision. This indicates that our method could only be used when the aggregate data have been obtained from a sufficient number of individuals and also when the residual variability is low. It is noted that, regarding acceptable bias and precision margins, although some rules of thumb are used in the literature (e.g., acceptable RMSE values between 0.2 and 0.5), there is no consensus on generally acceptable values for any of the criteria, %RBIAS, %RMSE, and %RAE, since they are better interpreted and applied comparatively rather than as absolute values. These criteria are used to interpret and compare across different models or datasets. In our case, they were utilized to compare across three different dataset scenarios.

Regarding residual variability, the proposed method could be criticized due to that it cannot distinguish it from IIV since there are no data in the individual level. This is, indeed, one of the weaknesses of the method, and the impact of that was studied above and was found to be significant only when residual variability was high. However, while in the framework of population pharmacokinetics, the residual variability is considered to be random noise added to the model predictions (and this includes sampling time error, measurement error, and variabilities other than IIV), in reality, it has several components, some of which are not random at all and are attributed to model misspecification [26]. In our methodology, model misspecification is accounted for in the residual error term sigma_1, which is the residual between the model predicted mean concentrations and the observed mean concentrations. Furthermore, our methodology allows to add a fixed random noise that could account for a known analytical error; however, this cannot be estimated. Therefore, residual variability is not ignored in our method.

It is also noted that in cases of very high residual variability, i.e., over 40%, then the proposed method is expected to produce more biased and less precise estimates of the PK parameters, especially regarding the IIV terms. Nevertheless, it should be noted that such high residual variability values could also indicate model misspecification, which should be taken into consideration in the analysis. Indeed, departure of the model from the data, which could contribute to the magnitude of residual unexplained variability in our methodology, is accounted for in the residual error of the means (term: sigma_1), as discussed above.

### 3.2. Application to the Real Datasets of Gevokizumab

#### 3.2.1. Dosage Group of 7 mg

As far as the application of our method to the real data of gevokizumab is concerned, the estimated PopPK parameters of the drug for the 7 mg dosage group are presented in Table 3. In this Table, the parameter names sigma_1 and sigma_2 correspond to the two residual error terms for the means and the SDs, respectively, as mentioned above. In addition, CLp_mean refers to mean plasma clearance, rc_1__mean refers to the mean reflection coefficient rc_1_, and rc_2__mean refers to the mean reflection coefficient rc_2_. Also, the parameter names ω_CLp_ and ω_V_ correspond to the two exponential IIV terms for plasma clearance and volume of human body, respectively.

The primary convergence statistic in Stan, for MCMC sampling, is the potential scale reduction factor (R_hat_) of Gelman and Rubin [27]. R_hat_ is computed for each scalar quantity of interest and is equal to the standard deviation of that quantity, from all the chains included together, divided by the root mean square of the separate within-chain standard deviations. Vehtari et al. have recently proposed modifications to R_hat_ on rank-normalizing and folding the posterior draws, only using the sample if R_hat_ is less than 1.01 for each parameter [28].

Also, as they note, a small value of R_hat_ is not enough to ensure that an MCMC sample is useful in practice. The effective sample size (ESS) must also be large enough to obtain stable inferences for quantities of interest. Roughly speaking, the ESS of a quantity of interest captures how many independent draws contain the same amount of information as the dependent sample obtained by the MCMC algorithm. The higher the ESS, the better. Vahteri et al. also recommend computing the ESS on the rank-normalized sample, as with R_hat_. Specifically, this computes the ESS of a sample from a rank-normalized version of the quantity of interest, using the rank transformation followed by the inverse normal transformation. To ensure reliable estimates of the variances and autocorrelations needed for R_hat_ and ESS, it is recommended that for each parameter, bulk-ESS as well as tail-ESS, crude measures of ESS for bulk and tail quantities, respectively, are greater than 400, when running four chains (default setting). An ESS greater than 100 per chain is considered good. This number was found to be typically sufficient to get a stable estimate of the Monte Carlo standard error (Vehtari et al., 2019) [28].

A GitHub repository (https://github.com/PMXathens/Gevokizumab, accessed on 12 June 2024) has been set up to host the source code. As far as our fit for the 7 mg dosage group is concerned, the related RData file named “ONE_DOSE.RData” is provided in the GitHub repository. In addition, a “7 mg dosage-group.R” file is provided, where after loading the previous RData file and executing the displayed commands, the values of bulk-ESS and tail-ESS are presented for each parameter. All these values are significantly greater than 400 for the four chains that we ran. Also, for each parameter, R_hat_ is less than 1.01. Moreover, traceplots, as shown in the Supplementary Materials (Figures S2–S8), were generated to graphically inspect convergence of the simulated Markov chains. The similar behavior for all four chains, the absence of patterns, as well as the absence of regions where the chains remain static suggest that convergence has been achieved. Finally, the HMC-related diagnostic tools indicated no pathological behavior.

**Figure 5:**
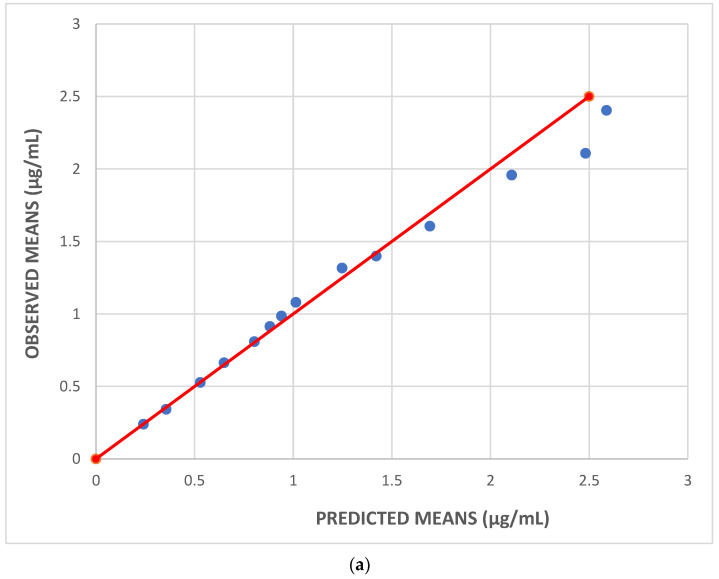

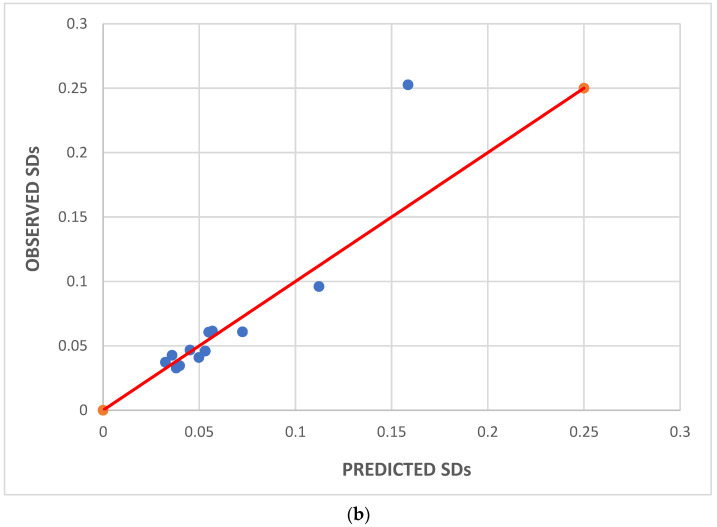
For the 7 mg dosage group: (**a**) observed versus predicted mean plasma concentrations of gevokizumab; (**b**) observed versus predicted SDs of plasma concentrations of gevokizumab. In both plots, the unit line is depicted in red.

Moreover, observed versus predicted plots of the mean plasma concentrations as well as their SDs are shown in Figure 5a (means) and Figure 5b (SDs). In both Figures, the dispersion of all values is symmetrical along the red unit line. It is also noted that the exponential error was used for both the means and the SDs, since mean plasma concentrations and their SDs are always positive. Hence, the dispersion is narrower at lower values and wider at higher values along the unit lines.

#### 3.2.2. Five Dosage Groups

Concerning the application of our method to the five datasets of gevokizumab, the estimated parameters of the drug for the different dosage groups are presented in Table 4. The parameter names sigma_1 and sigma_2 correspond to the two exponential residual error terms for the means and the SDs, respectively, CLp_mean refers to the mean group PopPK parameter CL_p_, rc_1__mean is the mean group PopPK parameter rc_1_, and rc_2__mean is the mean group PopPK parameter rc_2_. In addition, γ_CLp_, γ_rc1_, and γ_rc2_ are the IGV terms for CL_p_, rc_1_ and rc_2_, respectively. Furthermore, the parameter names ω_CLp_ [i] and ω_V_ [i] correspond to the two exponential IIV for plasma clearance and volume of human body, respectively, for the ith dataset (i = 1–5). Also, log_CLp [i], log_rc_1_ [i] and log_rc_2_ [i] are the logarithms of mean CL_p_, rc_1_ and rc_2_, respectively, for each one of the ith dataset.

An R file for the fit of all five dosage groups, named “FIVE_DOSES_FIT.R”, as well as a stan file, named “FIVE_DOSES_FIT”, are provided in the GitHub repository. The related RData file “FIVE_DOSES” is also provided. All the values of bulk-ESS and tail-ESS for each parameter are, again, more than 400 for the four chains that we ran. Moreover, for each parameter, R_hat_ is less than 1.01 and all the standard errors of the means are fairly low. Also, the HMC-related diagnostic tools indicated no pathological behavior and the traceplots that were generated suggest successful convergence.

It is noted that the estimated mean group PopPK parameters CLp and rc_1_ are close enough but not identical to the ones estimated by Cao et al. in 2013. More specifically, in this study, mean plasma clearance was estimated 0.0064 L/h, while Cao et al. found it was 0.00668 L/h, and the estimated value of the reflection coefficient rc_1_ in our study was 0.9504, while Cao et al. estimated it to be 0.931. However, the estimation of the reflection coefficient rc_2_ in our study (0.7674) seems to be moderately less than the one estimated by Cao et al. (0.837). Furthermore, the estimated values of IIV for plasma clearance (ω_CLp_ [i], with i = 1–5) as well as for volume of human body (ω_V_ [i], with i = 1–5) for each one of the dosage groups are reasonable but present a wide range. This wide range could be attributed either to the small number of patients, from which the aggregate data were obtained, or to potential differences among the dosage groups. The insertion of IGV in our method attempts to handle this issue. Another thing that must be noted is that the model for our last fit with the five dosage groups was run for 41,173.5 s. Hence, the cost of estimating the PopPK parameters from aggregate data, namely, data from which information has been removed, is the considerable consumption of time.

**Figure 6:**
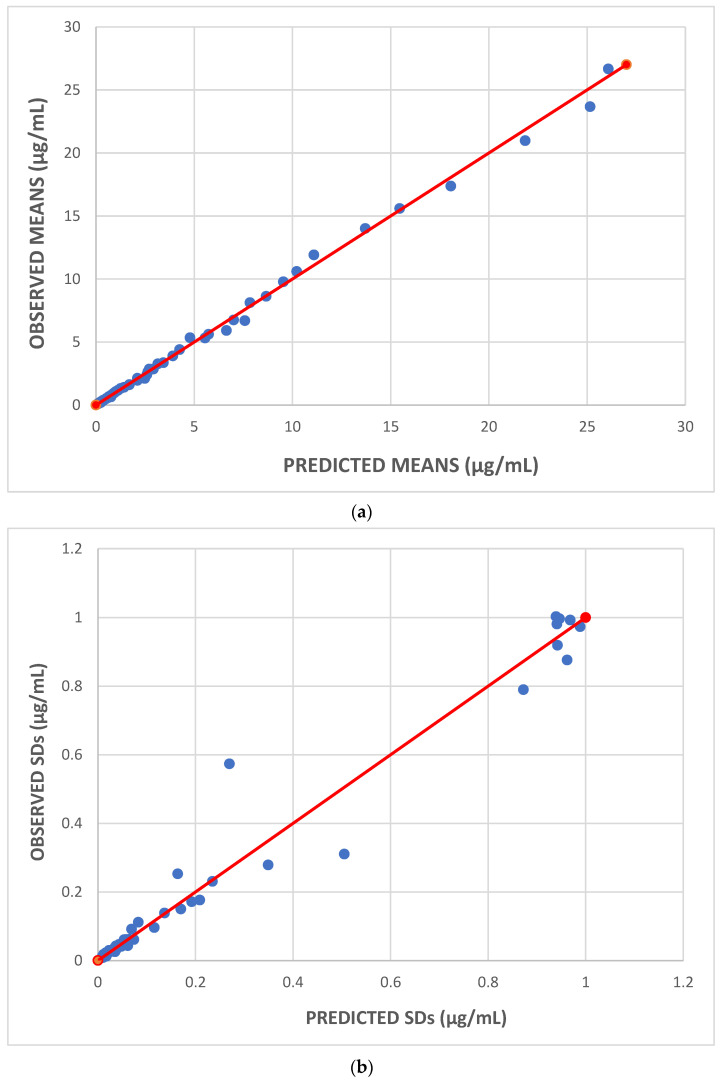
For all five dosage groups: (**a**): observed versus predicted mean plasma concentrations of gevokizumab; (**b**) observed versus predicted SDs of plasma concentrations of gevokizumab. In both plots, the unit line is depicted in red.

The observed versus predicted plots of the mean plasma concentrations and their SDs are shown in Figure 6a (means) and Figure 6b (SDs). Again, as shown in both Figures, the dispersion of all values is symmetrical along the red unit line. In addition, regarding mean plasma concentrations, the dispersion is narrower at lower values and wider at higher values along the unit line because of the exponential errors that were used. However, as far as the SDs are concerned, a few points deviate the most from the unit line although they do not have the highest values. For example, one such point in Figure 6b is the one with an observed SD value of 0.57 μg/mL and a predicted value of 0.27 μg/mL. Considering that the captured digitized SD values for the fourth dosage group, after an IV dose administration, were 0.31, 0.28, 0.57, 0.23, 0.18, and 0.17 μg/mL at the respective time points 24, 48, 72, 96, 168, and 216 h, we conclude that the unreasonable increase in a specific SD value (0.57 μg/mL) versus time indicates that this point is most likely an outlier.

It is also noted that in our method, only the three mean PK parameters (CL_p_, rc_1_, and rc_2_) for each one of the five groups were assumed to follow a Student’s t-distribution, which has heavier tails than the normal distribution and is used to down-weight outliers, while two different IIV terms (ω_CLp_ and ω_V_) were assumed for each group, as mentioned above, in a semi-hierarchical approach. It was decided that both of these terms should not follow a Student’s t-distribution, even though a prior of half-normal (0,1) was placed on them, since this was an excessive placement considering that the squares of the terms actually follow an inverse-gamma distribution. Despite this, our decision was based on our experience that a prior of half-normal (0,1) works better for these parameters. Therefore, our method is capable of down-weighting outliers concerning mean plasma concentrations but is possibly not able to down-weight outliers regarding SDs of plasma concentrations.

### 3.3. Application of the Method to a Variety of Drugs and PK Models

Our method could be applied not only to gevokizumab or monoclonal antibodies in general, but also to a variety of drugs and PK models. To highlight this, in the Supplementary Materials, a simple template is provided, while two related files, an R file named “iv_bolus” and a Stan file named “iv_bolus”, as well, are provided in the GitHub repository. As described in detail in the Supplementary Materials, a theoretical IV administration of a drug, following a simple one-compartment model, was utilized. Briefly, simulated aggregate data were generated and means along with SDs for 1000 virtual patients were predicted. Finally, for each time point, the predicted mean plasma concentrations and their corresponding SDs were fitted to the respective simulated aggregate data, providing good results.

Therefore, this template could be used as a guide when one analyst wishes to apply the proposed methodology to a drug of his/her interest with a more complicated PK model (two- or three- compartment model), when only aggregate data are available (mean plasma concentrations and their SDs). However, it is noted that there are certain limitations of the current methodology. For example, although the proposed method has been and could be applied to IV data, oral data should be handled with much more caution given the flip-flop phenomenon that could be present within the PK analysis. Also, when the observed aggregate data are obtained from few people, it is very difficult to predict accurate popPK parameters, especially IIVs. Additionally, during the prediction of means and SDs in our method, residual variability is not added upon the patient’s concentrations. This could have a major impact in cases of high residual variability in the observed data. Despite this, our methodology should be applied only as a proxy for raw data in cases when merely aggregate data are available, for example, when data of an old drug are only provided in forms of graphs with means and SD error bars or when data of new, novel drugs are given in similar forms to conceal information and enhance confidentiality.

## 4. Conclusions

In the present study, a methodology to estimate PopPK parameters, along with IIVs, using only reported patients’ mean plasma concentrations and their SDs versus time is proposed, given that aggregate data of this form are often found in published graphs. Since individual raw data do not exist in such cases, a Bayesian solution is provided by utilizing virtual patients and using simulation to reconstruct the likelihood of the aggregate data. The method was assessed by its application to simulated as well as real data of gevokizumab to estimate the drug’s PopPK parameters of a second-generation mPBPK model. Also, a mixed effects approach was used to allow accounting for the potential IGV among the real data of the five dosage groups of gevokizumab.

Conclusively, the methodology described adequately the data of gevokizumab, indicating that the removal/loss of information from averaging can be recovered. This recovery may be time consuming, especially for PK models with many differential equations, but is still reachable with the use of LHS. In addition, the number of patients from whom the aggregate data have been obtained should be sufficient to have adequate inferences. Hence, the proposed method could be applied to various monoclonal antibodies, for which the second-generation mPBPK model has been proposed, as well as to drugs with different PK models to estimate their PopPK parameters, when only aggregate data are available. Moreover, this method could be utilized when multiple aggregate concentration-time datasets from different sources need to be combined in a Bayesian meta-analysis approach to estimate the PoPK parameters of drugs.

## Figures and Tables

**Table 1:** Parameter values used for the simulation study.

Parameters	Dataset I	Dataset II	Dataset III
N	24	24	10
rv (%)	5	10	5
Dose (mg)	7	7	7
CL_p_ (L/h)	0.00668	0.00668	0.00668
rc_1_	0.931	0.931	0.931
rc_2_	0.837	0.837	0.837
$\omega_{CLp}$ (%)	20	20	20
$\omega_{V}$ (%)	20	20	20

**Table 2:** Summary of the bias (%RBIAS) as well as the precision (%RMSE and %RAE) of the estimates for the three dataset scenarios considered in our simulation study.

	Dataset I (rv = 5%, N = 24)	Dataset II (rv = 10%, N = 24)	Dataset III (rv = 5%, N = 10)
Mean PopPK parameters			
rc_1_			
%RBIAS	0.05727019	0.3271362	−0.007274277
%RMSE	1.227154	1.588526	2.048936
%RAE	0.924102	1.293161	1.535167
rc_2_			
%RBIAS	−0.08333982	−0.2648748	−0.5783198
%RMSE	1.627635	2.211325	2.688598
%RAE	1.278485	1.754442	2.116847
CLp			
%RBIAS	−0.01624675	−0.4528178	0.07686021
%RMSE	0.5339084	1.005213	0.8498672
%RAE	0.4371228	0.805272	0.6663747
IIV terms			
ω_CLp_			
%RBIAS	0.7168948	9.489459	−0.341962
%RMSE	5.340278	12.17255	9.716216
%RAE	4.408732	10.12623	7.257112
ω_V_			
%RBIAS	5.636213	20.4582	4.685937
%RMSE	8.651427	22.61808	12.41132
%RAE	6.85914	20.4582	9.153764

**Table 3:** Posterior PopPK parameter estimates obtained for the 7 mg dosage group from gevokizumab’s real data. The parameter names are described in the text.

Parameter	Mean	SE_mean	SD	2.5%	50%	97.5%	N_eff_
sigma_1	0.0758	0.0004	0.019	0.049	0.072	0.123	1989.956
sigma_2	0.2316	0.0012	0.056	0.151	0.222	0.370	2133.417
CLp_mean	0.0065	0.0000	0.000	0.006	0.007	0.007	1157.409
rc_1__mean	0.9584	0.0011	0.033	0.877	0.966	0.999	967.447
rc_2__mean	0.7645	0.0008	0.031	0.709	0.762	0.830	1415.577
ω_CLp_	0.0775	0.0002	0.010	0.059	0.077	0.100	2162.507
ω_V_	0.0699	0.0001	0.007	0.057	0.070	0.084	2994.706

**Table 4:** Posterior PopPK parameter estimates obtained for all five dosage groups from gevokizumab’s real data. The parameter names are described in the text. Indices [1–5] correspond to the first, second, third, fourth, and fifth dosage group, respectively.

Parameter	Mean	SE_Mean	SD	2.5%	50%	97.5%	N_eff_
sigma_1	0.0734	0.0001	0.007	0.061	0.073	0.089	3739.926
sigma_2	0.2706	0.0005	0.033	0.215	0.268	0.343	4736.458
CLp_mean	0.0064	0.0000	0.000	0.006	0.006	0.007	910.859
rc_1__mean	0.9504	0.0006	0.025	0.895	0.954	0.990	1680.591
rc_2__mean	0.7674	0.0013	0.058	0.647	0.767	0.896	2126.448
γ_CLp_	0.1254	0.0029	0.101	0.029	0.102	0.368	1191.566
γ_rc1_	0.0338	0.0008	0.037	0.003	0.023	0.130	2152.609
γ_rc2_	0.1810	0.0029	0.125	0.054	0.148	0.496	1872.394
ω_CLp_ [1]	0.1813	0.0020	0.109	0.010	0.176	0.401	2872.073
ω_CLp_ [2]	0.1138	0.0010	0.049	0.011	0.118	0.201	2430.177
ω_CLp_ [3]	0.0798	0.0002	0.012	0.059	0.079	0.104	5461.348
ω_CLp_ [4]	0.0374	0.0002	0.009	0.022	0.037	0.056	2932.790
ω_CLp_ [5]	0.2002	0.0004	0.028	0.147	0.199	0.258	3961.537
ω_V_ [1]	0.1676	0.0003	0.021	0.123	0.168	0.207	4222.692
ω_V_ [2]	0.3213	0.0027	0.111	0.060	0.332	0.505	1657.621
ω_V_ [3]	0.0733	0.0001	0.008	0.058	0.073	0.090	3424.664
ω_V_ [4]	0.0960	0.0001	0.010	0.078	0.096	0.117	5452.132
ω_V_ [5]	0.1000	0.0010	0.045	0.008	0.105	0.181	1906.016
log_CLp [1]	−5.0572	0.0013	0.068	−5.205	−5.056	−4.928	2925.386
log_CLp [2]	−4.9818	0.0009	0.040	−5.060	−4.981	−4.903	2007.067
log_CLp [3]	−5.0418	0.0005	0.030	−5.101	−5.041	−4.985	4136.353
log_CLp [4]	−5.1067	0.0004	0.028	−5.162	−5.106	−5.054	4340.950
log_CLp [5]	−5.1280	0.0006	0.033	−5.197	−5.127	−5.065	3607.724
log_rc_1_ [1]	−0.0588	0.0008	0.037	−0.152	−0.052	−0.007	1944.272
log_rc_1_ [2]	−0.0528	0.0007	0.030	−0.123	−0.049	−0.007	1890.579
log_rc_1_ [3]	−0.0441	0.0006	0.026	−0.103	−0.041	−0.004	1731.245
log_rc_1_ [4]	−0.0489	0.0006	0.026	−0.106	−0.046	−0.006	1789.105
log_rc_1_ [5]	−0.0513	0.0006	0.028	−0.115	−0.047	−0.007	2002.731
log_rc_2_ [1]	−0.4421	0.0011	0.055	−0.544	−0.444	−0.326	2731.710
log_rc_2_ [2]	−0.2947	0.0009	0.043	−0.378	−0.295	−0.210	2546.313
log_rc_2_ [3]	−0.2639	0.0007	0.035	−0.330	−0.266	−0.194	2263.931
log_rc_2_ [4]	−0.1878	0.0006	0.031	−0.245	−0.189	−0.123	2316.835
log_rc_2_ [5]	−0.2086	0.0007	0.035	−0.273	−0.210	−0.140	2365.040

## Data Availability

The original contributions presented in the study are included in a public GitHub repository, https://github.com/PMXathens/Gevokizumab, accessed on 12 July 2024. further inquiries can be directed to the corresponding author.

## Supplementary Materials

The following supporting information can be downloaded at: https://www.mdpi.com/article/10.3390/pharmaceutics16091129/s1, Description of mPBPK model, Template, Traceplots for the 7 mg dosage-group. Figure S1: Second-generation minimal PBPK model for gevokizumab pharmacokinetics; Figure S2: Traceplot for sigma_1; Figure S3: Traceplot for sigma_2; Figure S4: Traceplot for CLp_mean; Figure S5: Traceplot for rc1_mean; Figure S6: Traceplot for rc2_mean; Figure S7: Traceplot for ω_CLp_; Figure S8: Traceplot for ω_V_.
