# Supplementary material for: A Meta-Analysis Methodology in Stan to Estimate Population Pharmacokinetic Parameters from Multiple Aggregate Concentration–Time Datasets: Application to Gevokizumab mPBPK Model
Source: Pharmaceutics. 2024 Aug 27;16(9):1129. doi: 10.3390/pharmaceutics16091129 (PMC11434912; doi:10.3390/pharmaceutics16091129)
Supplement: Supplementary file 1 [file pharmaceutics-16-01129-s001.zip › pharmaceutics-3129620-supplementary.pdf]

A Meta-Analysis Methodology in Stan to Estimate Population Pharmacokinetic Parameters from Multiple Aggregate Concentration–Time Datasets: Application to Gevokizumab mPBPK Model

Evangelos Karakitsios and Aristides Dokoumetzidis *

Department of Pharmacy, National and Kapodistrian University of Athens, Panepistimiopolis Zografou, 15784 Athens, Greece

Supplementary Material

mPBPK model

Differential equations for second generation mPBPK model for gevokizumab

$$\frac{dC_p}{dt} = \frac{Input}{V_p} + \left[C_{lymph} \cdot L - C_p \cdot L_1 \cdot (1 - rc_1) - C_p \cdot L_2 \cdot (1 - rc_2) - C_p \cdot CL_p\right]/V_p \quad (S1)$$

$$\frac{dC_{tight}}{dt} = \left[L_1 \cdot (1 - rc_1) \cdot C_p - L_1 \cdot (1 - rc_L) \cdot C_{tight}\right]/V_{tight} \quad (S2)$$

$$\frac{dC_{leaky}}{dt} = \left[L_2 \cdot (1 - rc_2) \cdot C_p - L_2 \cdot (1 - rc_L) \cdot C_{leaky}\right]/V_{leaky} \quad (S3)$$

$$\frac{dC_{lymph}}{dt} = \left[L_1 \cdot (1 - rc_L) \cdot C_{tight} + L_2 \cdot (1 - rc_L) \cdot C_{leaky} - C_{lymph} \cdot L\right]/V_{lymph} \quad (S4)$$

where C_p and C_{lymph} are antibody concentrations in plasma and lymph respectively. In addition, C_{tight} and C_{leaky} are antibody Interstital Fluid (ISF) concentrations in tissues with continuous endothelium and with fenestrated or discontinuous endothelium, respectively, V_{tight} is volume of ISF in tight tissues and V_{leaky} is volume of ISF in leaky tissues. Also, L is total lymph flow and equals the sum of L_1 and L_2, where $L_1 = 0.33*L$ and $L_2 = 0.67*L$. The terms rc_1 and rc_2 are the respective vascular reflection coefficients for V_{tight} and V_{leaky}. It is noted that these coefficients are parameters that not only determine transcapillary rate but also predict the extent of distribution. The lower vascular reflection coefficient produces a more rapid transcapillary rate, resulting in earlier peaking and higher concentrations of the monoclonal antibody in the lumped ISF compartment.
Furthermore, V_p is plasma volume, V_{lymph} is total lymph volume, rc_L is the lymphatic capillary reflection coefficient, which is assumed to be 0.2, while CL_p is plasma clearance. In addition, Input is the amount of drug taken initially and all initial conditions are concentrations = 0. The physiological restrictions of the model are shown in equations (S5) - (S8):

$$rc_1 < 1 \quad (S5)$$

$$rc_2 < 1 \quad (S6)$$

$$V_{tight} = 0.65 \cdot V_{ISF} \cdot K_p \quad (S7)$$

$$V_{leaky} = 0.35 \cdot V_{ISF} \cdot K_p \quad (S8)$$

where V_{ISF} is total system volume of ISF and K_p is available fraction of ISF for antibody distribution. For gevokizumab Kp is equal to 0.8. Lastly, the physiologic parameters for a 70 kg body weight person are: L = 2.9 L/day, V_{ISF} = 15.6 L, V_{lymph} = 5.2 L and V_{plasma} = 2.6 L (Cao et al., 2013).

The model structure of this second generation mPBPK is shown in the following Figure S1:

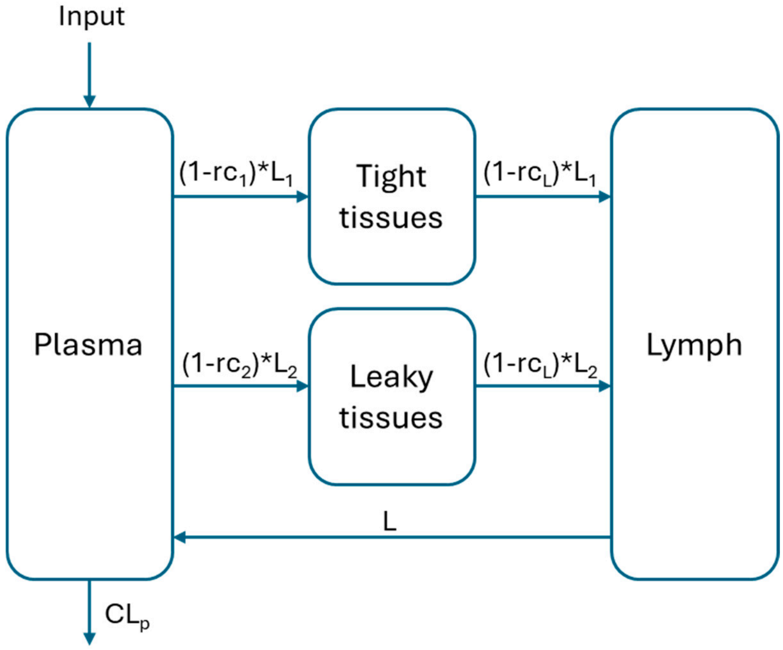

Figure S1: Second-generation minimal PBPK model for gevokizumab pharmacokinetics. Symbols and physiological restrictions are defined in equations (S1) - (S4). The plasma compartment in the left box represents venous plasma as in full, whole-body PBPK models but is not applied in this model. The plot is adapted from the study of Cao et al., in 2013.

Template

To emphasize that our methodology could be applied to a variety of drugs with different PK models, a simple template is provided. In this template, initially simulated aggregate data from 24 patients were generated of a theoretical drug that was assumed to be administered with a plain IV bolus (one compartment model – single dose). More particularly, the values of drug plasma clearance (CL) and volume of distribution (V_d) for these 24 patients were assumed to follow a lognormal distribution. The parameters for the simulation were fixed as follows: mean plasma clearance was 5 L/h, mean volume of distribution was 20 L and the SDs of the lognormal distributions of plasma clearance as well as of volume of distribution of these patients were 20%. In addition, the dose was 100mg, while the time points, when plasma concentrations were assumed to be measured were 1, 2, 4, 6, 8, 12, 16 and 24 hours. Then, the plasma concentrations for each one of the 24 patients were calculated according to equation (S9). Afterwards, an exponential residual variability term equal to 0.05 was assumed for every individual's concentration at each time point and, lastly, the mean plasma concentrations as well as their SDs were obtained. This is the simulated data.

$$Cp = \frac{D}{Vd} * e^{-\frac{CL}{Vd} * t} \qquad \text{(S9)}$$

where Cp is plasma concentration, D refers to the Dose and t are the time points.

The same procedure was also followed in Stan to obtain the predicted means and SDs for 1000 virtual patients, apart from the fact that residual variability was not added to the patients' concentrations. In particular, the model was parametrized in terms of drug plasma clearance, volume of distribution as well as the two IIV terms, for the SD of the lognormal distributions of plasma clearance and volume of distribution. Also, two separate exponential residual error terms were assumed, one for the means and one for the SDs. The Bayesian priors used for the model's parameters were as less informative as possible. Finally, for each time point the predicted mean plasma concentrations and their corresponding SDs were fitted to the respective simulated aggregate data. It is noted that in this particular case the target acceptance rate adapt_delta was increased, as suggested by Stan's warning message, since there were divergent transitions after warmup.

Therefore, this template, concerning a simple IV bolus administration, could be used as a guide when one analyst wishes to apply our methodology to a drug of his/her interest with a much more complicated PK model.

Traceplots for the 7kg dosage-group

The following Figures depict the traceplots for the parameters used in the 7kg dosage-group. These parameters include sigma_1 (Figure S2), sigma_2 (Figure S3), CLp_mean (Figure S4), rc_1_mean (Figure S5), rc_2_mean (Figure S6), ω_{CLp} (Figure S7) and ω_V (Figure S8). In each Figure the 4 chains are depicted with a different color (chain 1: red; chain 2: blue; chain 3: purple; chain 4: yellow).

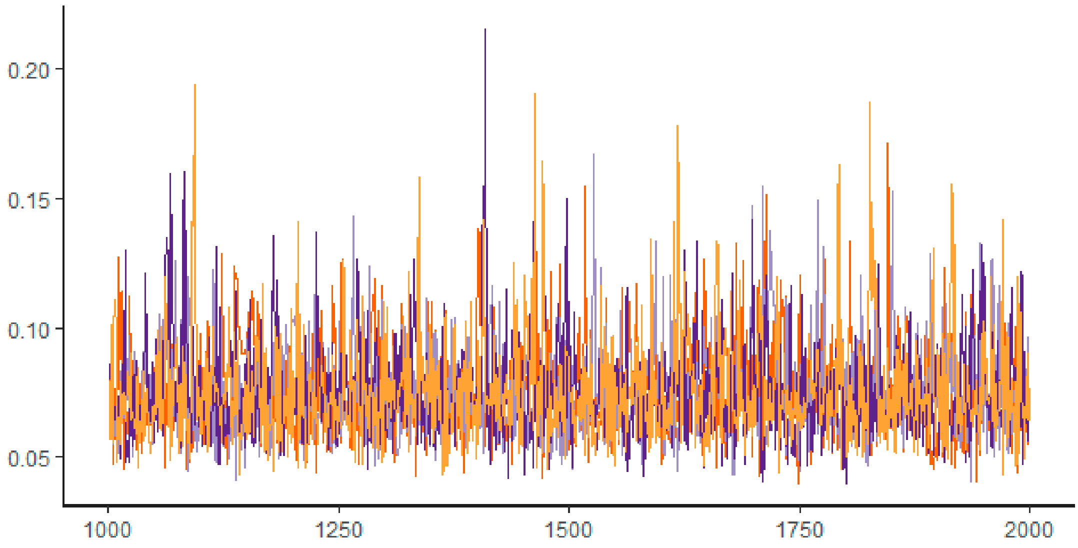

Figure S2: Traceplot for sigma_1

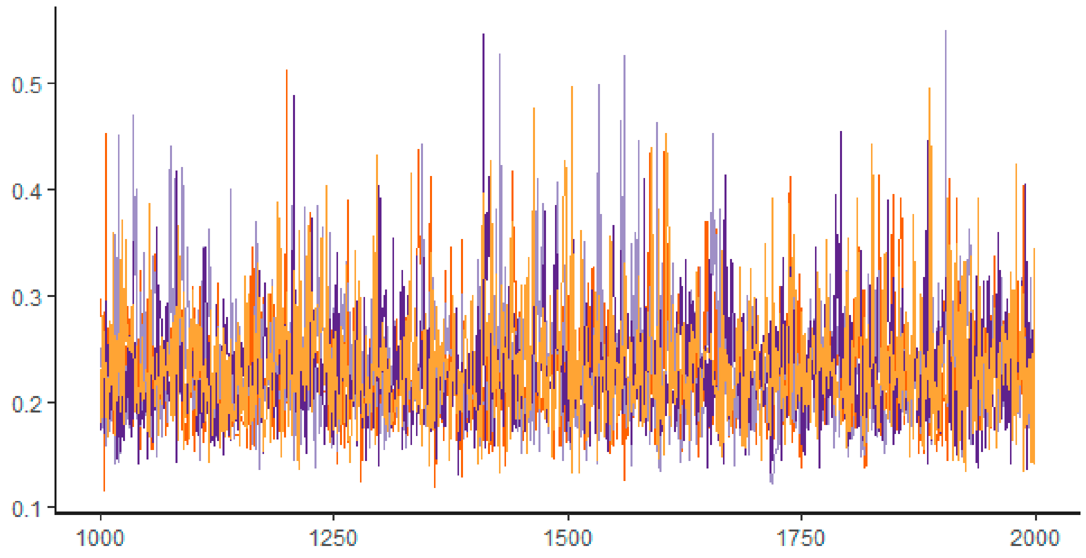

Figure S3: Traceplot for sigma_2

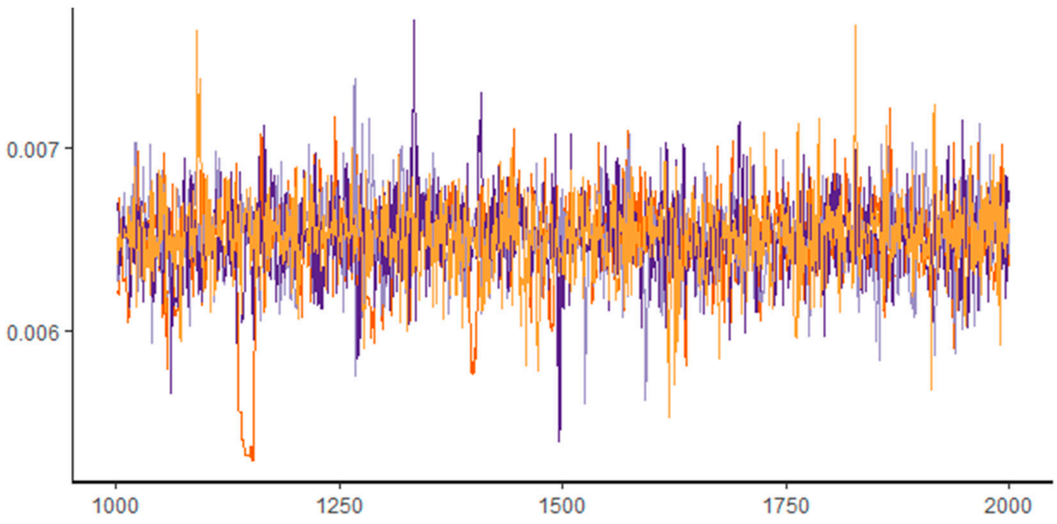

Figure S4: Traceplot for CLp_mean

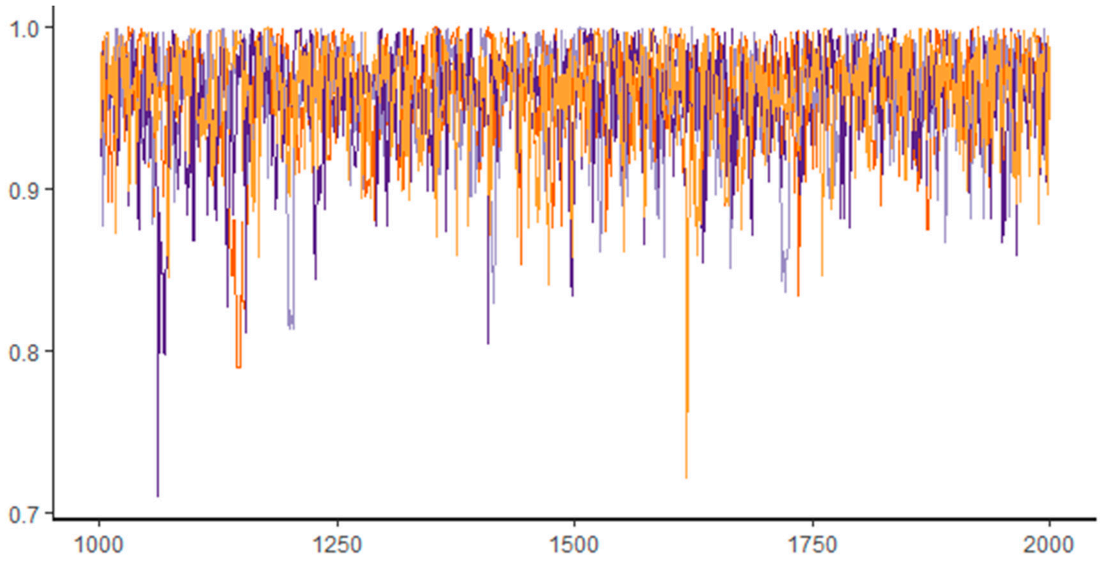

Figure S5: Traceplot for rc_1_mean

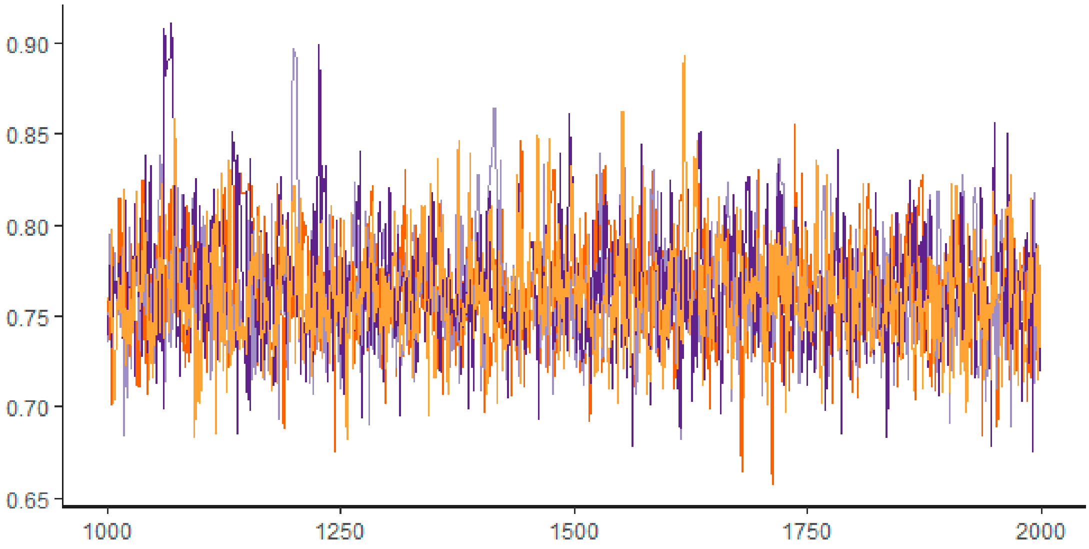

Figure S6: Traceplot for rc_2_mean

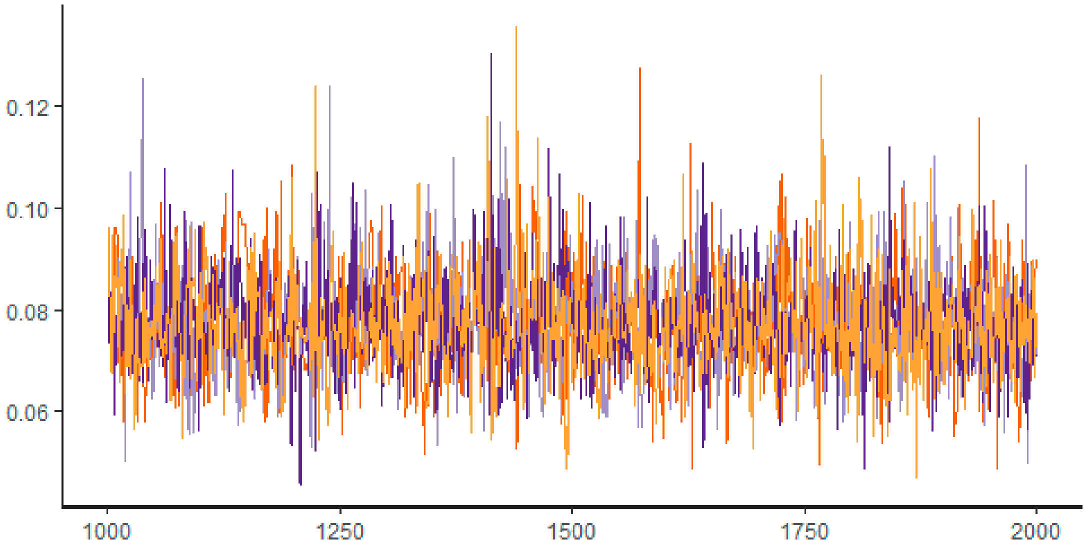

Figure S7: Traceplot for ω_{CLp}

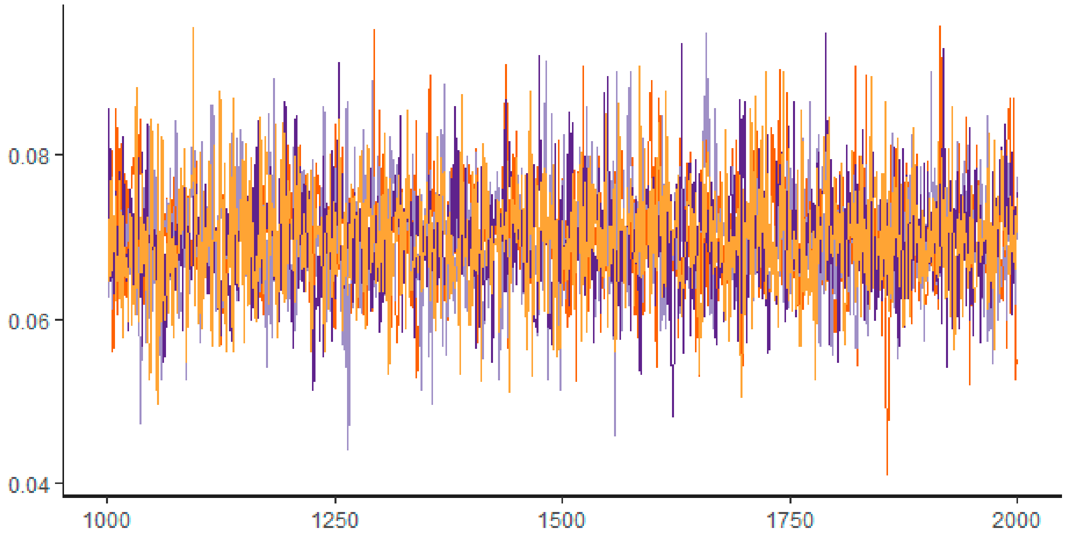

Figure S8: Traceplot for ω_{V}
